# Assembly and Analysis of the Mitochondrial Genome of *Hippophae rhamnoides* subsp. *sinensis*, an Important Ecological and Economic Forest Tree Species in China

**DOI:** 10.3390/plants14142170

**Published:** 2025-07-14

**Authors:** Jie Li, Song-Song Lu, Yang Bi, Yu-Mei Jiang, Li-Dan Feng, Jing He

**Affiliations:** 1 College of Forestry, Gansu Agricultural University, Lanzhou 730070, China; lj81658@gsau.edu.cn (J.L.); hejing268@aliyun.com (J.H.); 2 Wolfberry Harmless Cultivation Engineering Research Center of Gansu Province, Lanzhou 730070, China; 3 College of Food Science and Engineering, Gansu Agricultural University, Lanzhou 730070, China; biyang@gsau.edu.cn (Y.B.); jym316@gsau.edu.cn (Y.-M.J.); fengld@gsau.edu.cn (L.-D.F.)

**Keywords:** *Hippophae. rhamnoides* subsp. *sinensis*, mitochondrial genome, repetitive sequences, homologous fragments, genetic evolution

## Abstract

*Hippophae rhamnoides* subsp. *sinensis* is extensively found in China, where the annual precipitation ranges from 400 to 800 mm. It is the most dominant species in natural sea buckthorn forests and the primary cultivar for artificial ecological plantations. Additionally, it exhibits significant nutritional and medicinal value, making it a renowned eco-economic tree species. Despite extensive research into its ecological functions and health benefits, the mitochondrial genome of this widespread species has not yet been published, and knowledge of the mitochondrial genome is crucial for understanding plant environmental adaptation, evolution, and maternal inheritance. Therefore, the complete mitochondrial genome was successfully assembled by aligning third-generation sequencing data to the reference genome sequence using the Illumina NovaSeq 6000 platform and Nanopore Prometh ION technologies. Additionally, the gene structure, composition, repeat sequences, codon usage bias, homologous fragments, and phylogeny-related indicators were also analyzed. The results showed that the length of the mitochondrial genome is 454,489 bp, containing 30 tRNA genes, three rRNA genes, 40 PCGs, and two pseudogenes. A total of 411 C-to-U RNA editing sites were identified in 33 protein-coding genes (PCGs), with higher frequencies observed in *ccmFn*, *ccmB*, *nad5*, *ccmC*, *nad2*, and *nad7* genes. Moreover, 31 chloroplast-derived fragments were detected, accounting for 11.86% of the mitochondrial genome length. The *ccmB*, *nad4L*, and *nad7* genes related to energy metabolism exhibited positive selection pressure. The mitochondrial genome sequence similarity between *H. rhamnoides* subsp. *sinensis* and *H. tibetana* or *H. salicifolia* was 99.34% and 99.40%, respectively. Fifteen shared gene clusters were identified between *H. rhamnoides* subsp. *sinensis* and *H. tibetana*. Phylogenetically, the Rosales order showed close relationships with Fagales, Fabales, Malpighiales, and Celastrales. These findings provide fundamental data for exploring the widespread distribution of *H. rhamnoides* subsp. *sinensis* and offer theoretical support for understanding the evolutionary mechanisms within the *Hippophae* genus and the selection of molecular breeding targets.

## 1. Introduction

The genus *Hippophae*, belonging to the family Elaeagnaceae, comprises six species and thirteen subspecies. Among these, *H. rhamnoides* subsp. *sinensis* is a subspecies of *H. rhamnoides*, exhibiting growth forms that include deciduous trees, small trees, or shrubs [1]. Renowned for its exceptional drought tolerance [2], salt–alkali resistance [3], and wind–sand erosion resistance, it is widely employed as a soil and water conservation species [4]. Additionally, its berries are rich in vitamin C [5], while its seed oil possesses notable antioxidant and anti-inflammatory properties [6], and its fruit oil contains abundant carotenoids [7], conferring high nutritional and health benefits. These attributes solidify its status as a prominent eco-economic tree species. China hosts the world’s largest expanse of sea buckthorn forest, covering approximately 2.26 million hectares, accounting for 88% of the global forest [8]. This includes 1.53 million hectares of plantations and 0.72 million hectares of natural forests, with an annual fruit yield ranging from 650,000 to 750,000 tons. *H. rhamnoides* subsp. *sinensis* thrives optimally in regions with an annual precipitation range of 441–782 mm, forming the dominant component of China’s sea buckthorn forest and serving as the primary species for artificial ecological plantations. Zhang et al. indicated that its highly suitable habitat extends up to 471,000 km^2^, underscoring its vast developmental potential [9].

Mitochondria are pivotal organelles in eukaryotic cellular energy metabolism [10,11]. Plant mitochondrial genome rearrangements serve as key drivers for accelerated molecular evolution, cytoplasmic male sterility, and developmental abnormalities [12]. Compared to their animal counterparts, plant mitochondrial genomes exhibit distinct characteristics, including large sizes, frequent rearrangements, high rates of inversions, and extensive recombination events [13]. Their size varies widely, ranging from 42 kb in *Mesostigma viride* [14] to 11.7 Mb in *Silene conica* [15]. A key driver of structural and size variation in plant mitogenomes is the ongoing and dynamic transfer of genes from chloroplasts and the nucleus into the mitochondrial genome [12,16]. Due to these complexities, studying plant mitogenomes poses significant challenges. However, advances in sequencing and assembly technologies have greatly enhanced the ability to accurately detect and assemble plant mitogenomes [16,17]. Comparative analyses of plant mitochondrial genomes have emerged as a crucial approach for investigating evolutionary mechanisms and identifying potential target genes plant breeding.

Between 2024 and 2025, the mitochondrial genome assemblies of several sea buckthorn species including *H. tibetana* [18], *H. salicifolia* [19] and *H. gyantsensis* [20] were completed. Furthermore, sequencing data for *H. rhamnoides*, *H. gyantsensis* and *H. tibetana* genomes were made available [21,22,23], offering important references and establishing a solid basis for this research. However, it should be noted that *H. tibetana*, *H. salicifolia* and *H. gyantsensis* have relatively limited geographical distributions. The chloroplast genome of *H. rhamnoides* subsp. *sinensis*, a species widely distributed in China, was published in 2020 [24], but its mitochondrial genome remains unreported. In this study, we assembled the complete mitochondrial genome and analyzed it. Our work includes an analysis of the characterization of gene structure, composition, codon usage bias, RNA editing and repetitive sequences. Furthermore, we explored homologous fragments between the mitochondria and chloroplasts, conducted Ka/Ks ratio analysis, and examined phylogenetic relationships among different species. These comprehensive analyses provide deeper insights into *H. rhamnoides* subsp. *sinensis*, revealing the reason for wide distribution.

## 2. Materials and Methods

### 2.1. Plant Materials, Mitochondrial DNA Isolation and Genome Sequencing

Fresh young leaves of *H. rhamnoides* subsp. *sinensis* were collected from a female tree (Supplementary Figure S1A,B) growing in Anning County, Lanzhou City, Gansu Province, China (103°42′18.71″ E, 36°5′45.28″ N). Leaves were quickly frozen in liquid nitrogen and then stored at −80 °C prior to DNA isolation.

The second-generation sequencing total genomic DNA was extracted using a plant genomic DNA kit DP305 (Tiangen Biotech, Beijing, China), and the third-generation sequencing total genomic DNA was extracted using CTAB method. The absorbance ratio OD260/OD280 of DNA solution used for second-generation sequencing should be between 1.7 and 1.9, and the ratio for third-generation sequencing should be between 1.8 and 2.0. DNA purity was detected with 1.0% agarose gel. To obtain the full-length mitochondrial genome with high accuracy, short-read and long-read sequencing technologies were combined in this study by Nanjing Genepioneer Technology Co., Ltd. (Nanjing, China). The Hieff NGS DNA Library Prep Kit (Yeasen, Shanghai, China) was used for second-generation sequencing library preparation, and the Ligation Sequencing Kit 1D SQK-LSK109 (Nanopore, Oxford, UK) was used for third-generation sequencing library preparation. The short-read sequencing platform was Illumina Novaseq 6000 (Illumina, San Diego, CA, USA) and the paired end sequencing (PE) read length was 150 bp, using the fastp '”v 0.20.0, https://github.com/OpenGene/fastp (accessed on 10/1/2022)“ software to filter the original data and obtain high-quality reads. The long-read sequencing platform was Nanopore Prometh ION (Nanopore, Oxford, UK), and then the sequencing data were filtered by filtlong “v0.2.1, https://link.zhihu.com/?target=https%3A//github.com/rrwick/Filtlong (accessed on 10/1/2022)“ software. The BaseSum was 6,769,252,800 bp in the second-generation sequencing. The number of bases was 9,538,144,619 bp, and the N50 read length was 27,484 bp in the third-generation sequencing.

To ensure the accuracy and reliability of subsequent bioinformatics analyses, we performed error correction using the software LoRDEC (v0.3) [25], which leverages second-generation sequencing data to polish the third-generation sequencing reads.

### 2.2. Assembly and Annotation of the Mitogenome

The third-generation comparison software Minimap2 (v2.1) [26] was used to compare the original third-generation data with the reference gene sequence (plant mitochondrial core gene) and screen for a sequence with a length greater than 50 bp as the candidate sequence in the alignment. A sequence with more aligned genes (one sequencing sequence contains multiple core genes) and higher alignment quality (covering more complete core genes) was selected as the seed sequence. By comparing the original long-read sequencing data with the seed sequence, sequences with a minimum overlap of 1 kb and at least 70% similarity were added to the seed sequence, and the original data were iteratively aligned to the seed sequence, so as to obtain all long-read sequencing data of the mitogenome. Then, the third-generation assembly software canu (v2.2) [27] was used to correct the third-generation data obtained, and Bowtie2 (v2.3.5.1) [28] was used to align the second-generation data to the corrected sequence. The default parameter Unicycler (v0.4.8) was used to compare the above second-generation data and the corrected third-generation data for concatenation. Finally, the ringed *H. rhamnoides* subsp. *sinensis* mitogenome was obtained, and the average depth of assembled mitogenomes was 325×.

Mitogenome structure annotation was performed using the following steps: (1) The encoded proteins and rRNAs were compared to published plant mitochondrial sequences using BLAST, and further manual adjustments were made based on closely related species; (2) The tRNA was annotated using tRNA scan SE “http://lowelab.ucsc.edu/tRNAscan-SE (accessed on 11/1/2022)“with default settings; (3) ORFs were annotated using Open Reading Frame Finder “http://www.ncbi.nlm.nih.gov/gorf/gorf.html (accessed on 11/1/2022)“, the shortest length was set to 102 bp, and redundant sequences and sequences overlapping with known genes were excluded. Sequence alignments greater than 300 bp were annotated to the NR library. The circular mitochondrial map was drawn using the Draw Organelle Genome Maps online software “OGDRAW v1.3.1, https://chlorobox.mpimp-golm.mpg.de/OGDraw.html (accessed on 11/1/2022)“. The generated .fsa and .tbl files were submitted to the NCBI database, from which we obtained the accession number PV762916 for *H. rhamnoides* subsp. *sinensis*.

### 2.3. Codon Usage Analysis

Relative Synonymous Codon Usage (RSCU) is thought to result from a combination of natural selection, mutation and genetic drift, and its numerical value is the ratio of the actual frequency of codon usage to the theoretical frequency of codon usage. A script written in Perl was used to filter unique CDSs (by choosing one from multiple copies of each CDS) and perform the calculations.

### 2.4. RNA Editing Analyses

The editing sites in the mitochondrial RNA of *H. rhamnoides* subsp. *sinensis* were identified using the mitochondrial gene-encoding proteins of plants as reference proteins. The analysis was conducted using the Plant Predictive RNA Editor (PREP, prediction thresholds 0.2) suite [29] (http://prep.unl.edu/).

### 2.5. Analysis of Repeat Sequences

Three kinds of repeats (simple sequence, tandem, and dispersed) were detected in the mitogenome. Simple repetitive sequence analysis was performed using MISA online software (v2.1) [30] “https://webblast.ipk-gatersleben.de/misa/, parameter:1-10 2-5 3-4 4-3 5-3 6-3 (accessed on 8/4/2022)“. In this analysis, we identified 10, 5, 4, 3, 3, and 3 repeats with 1, 2, 3, 4, 5, and 6 bases, respectively. Tandem repeats with lengths >6 bp and >95% matching repeat units were detected using Tandem Repeats Finder v4.09 software [31] “http://tandem.bu.edu/trf/trf.submit.options.html (accessed on 8/4/2022)“. The parameters were as follows: 2.7.7/80/10/50/2000 -f -d -m. Dispersed repeats were detected using BLASTN (v2.10.1, parameters: -word size 3, e-value 1 × 10^−5^, remove redundancy, remove tandem duplication). Circos v0.69-5 “http://circos.ca/software/download/ (accessed on 10/1/2022)“was used to visualize these repeats. For comparative analysis, we compared the dispersed repeats in the mitochondrial genome of *H. rhamnoides* subsp. *sinensis* with those in the published mitochondrial genomes of multiple species including *Astragalus membranaceus*, *Caragana spinosa*, *Glycyrrhiza glabra*, *Medicago sativa*, *Oxytropis arctobia*, *Pisum fulvum*, *Trigonella foenum-graecum*, and *H. tibetana*. The comparative results are shown in Table 1.

### 2.6. Ka/Ks Ratio Evaluation

To analyze synonymous and nonsynonymous substitution rates, we selected representative species from five species within the Rosales order, including three species from the genus *Hippophae*. The selected species were *H. gyantsensis* (PP314236.1), *H. tibetana* (PP712939), *H. salicifolia* (PQ653489), *Ziziphus jujuba* (NC_029809), and *Fragaria orientalis* (NC_057524) Homologous protein sequences between *H. rhamnoides* subsp. *sinensis* (PV762916) and those in the mitochondrial genomes of other species were identified using BLASTN v. 2.10.1 [32], and shared PCGs were aligned using MAFFT v. 7.310 [33]. Ka/Ks values, which measure the ratio of nonsynonymous to synonymous substitutions, were calculated using the MLWL model in Ka/Ks Calculator v. 2.0. (https://sourceforge.net/projects/kakscalculator2/ (accessed on 23/4/2025)“ [34].

### 2.7. Homologous Fragment Analysis

The chloroplast genome sequence of *H. rhamnoides* subsp. *sinensis* (NC_049156) was downloaded from the NCBI Organelle Genome Resources Database. BLAST software (v 2.13.0) on NCBI was used to identify the homologous fragments between the mitogenome and chloroplast genome. Screening criteria were set as matching rate ≥ 70%, e-value ≤ 1 × 10^−5^, and length ≥ 30 bp. The results were visualized using circos (v0.69-5).

### 2.8. Phylogenetic Tree Construction

To acquire the phylogenetic position of *H. rhamnoides* subsp. *sinensis*, 36 plant mitogenomes (Supplementary Table S1) were downloaded from the NCBI Organelle Genome Resources Database (http://www.ncbi.nlm.nih.gov/genome/organelle/ (accessed on 20/11/2024)“. Among these species, not only were the complete mitogenome sequences of these species for analysis available in NCBI, but they were also placed clearly in taxonomy and were widely used. The shared 28 CDSs from different species were aligned using MAFFT (v7.427, auto mode) software [33]. A maximum likelihood (ML) phylogenetic tree was constructed using RA×ML v8.2.10 [35] “https://cme.h-its.org/exelixis/software.html (accessed on 20/11/2024)” software, with the GTRGAMMA model and bootstrap = 1000. The optimal nucleotide substitution model was calculated using jModelTest v2.1.10 “https://github.com/ddarriba/jmodeltest2 (accessed on 20/11/2024)”, and then MrBayesv3.2.7a “http://nbisweden.github.io/MrBayes/ (accessed on 20/11/2024)” was used to establish a Bayesian inference phylogenetic tree. The parameters for MrBayes v3.2.7 software are based on jModelTest v2.1.10 results.

## 3. Results

### 3.1. Mitochondrial Genome of H. rhamnoides subsp. sinensis and Its Characteristics

**Figure 1:**
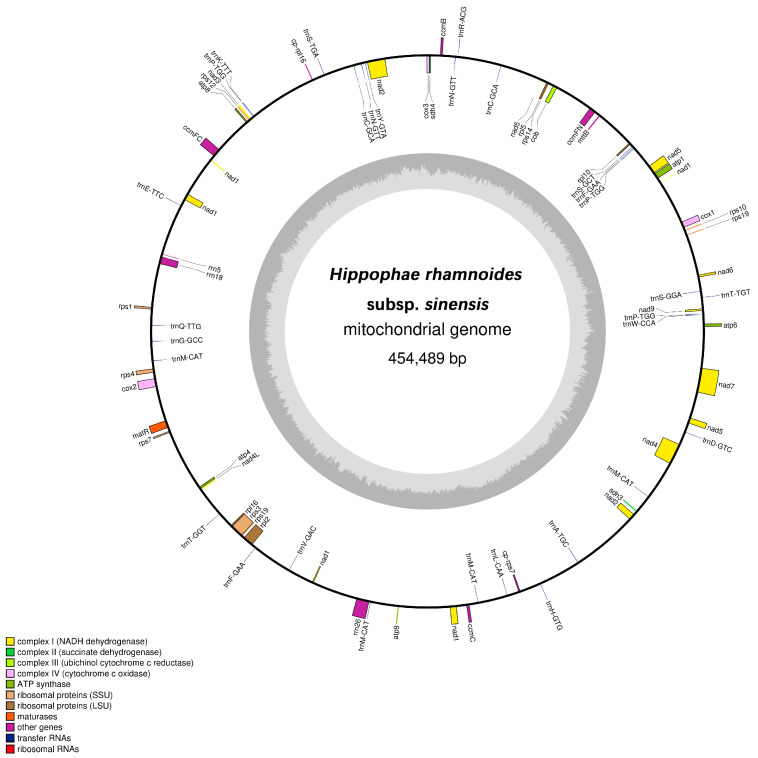
Mitochondrial genome map of *H. rhamnoides* subsp. *sinensis*. Note: Coding genes transcribed in the forward direction are displayed on the outer rim of the circle, whereas those transcribed in the reverse direction are presented on the inner rim. The innermost gray circle indicates the GC content across the genome.

According to the assembly protocol of the *H. rhamnoides* subsp. *sinensis* mitochondrial genome, a complete mitochondrial genome was successfully assembled, with a total length of 454,489 bp (Figure 1). The genome exhibited a GC content of 44.86%, indicating an AT-biased nucleotide composition. In terms of topological structure, the mitochondrial genome of *H. rhamnoides* subsp. *sinensis* adopted a master circle conformation.

The mitochondrial genome of *H. rhamnoides* subsp. *sinensis* encoded 75 annotated functional genes, including 30 tRNA genes, 3 rRNA genes, 40 PCGs, and 2 pseudogenes (Supplementary Table S2). Among the 40 PCGs, 26 were mitochondrial core genes, comprising 5 ATP synthase genes, 10 NADH dehydrogenase genes, 4 cytochrome c biogenesis genes, 3 cytochrome c oxidase genes, 1 transport membrane protein gene, 1 maturase gene, 1 ubiquinol cytochrome c reductase gene, and 1 succinate dehydrogenase gene. Additionally, there were 33 non-core mitochondrial genes, including 3 large subunit ribosomal protein genes and 30 small subunit ribosomal protein genes.

### 3.2. Codon Usage Bias in Mitochondrial Genome

Codon usage varies significantly among different species. We calculated the Relative Synonymous Codon Usage (RSCU) to investigate the codon usage bias in the mitochondrial genome of *H. rhamnoides* subsp. *sinensis*. In the complete mitochondrial genome of *H. rhamnoides* subsp. *sinensis*, a total of 10,551 codons were identified across 40 PCGs (Supplementary Table S3). The genome contains 64 standard codons, of which 61 sense codons encode all 20 amino acids, while the remaining 3 serve as translation termination signals.

**Figure 2:**
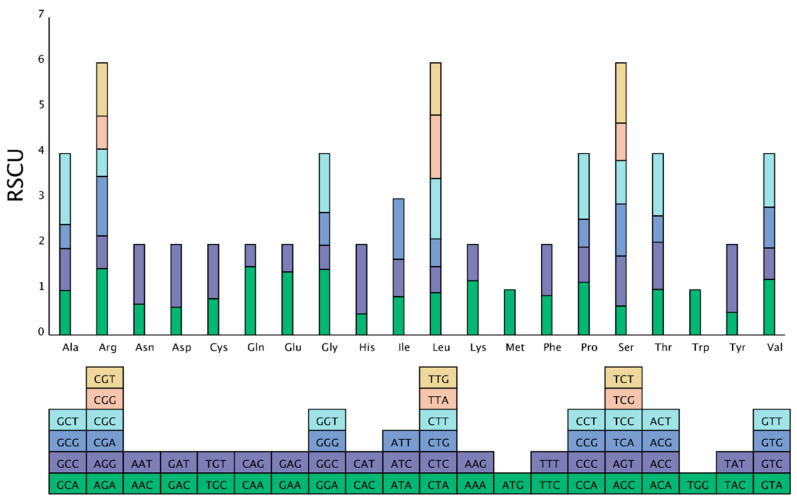
RSCU in the *H. rhamnoides* subsp. *sinensis* mitogenome. Note: The *x*-axis represents the type of amino acid. The *y*-axis represents the RSCU value. Each amino acid is encoded by multiple codons, which is shown by the histogram with different colors.

Codons with an RSCU value greater than 1 were defined as high-frequency codons, indicating preferential usage in amino acid encoding. For most amino acids, the codon usage exhibited bias, except for UGG (Trp, Tryptophan) and AUG (Met, Methionine) (Figure 2, Supplementary Table S3). A total of 32 high-frequency codons were identified in the mitochondrial genome of *H. rhamnoides* subsp. *sinensis*. For example, the termination codon UAA showed the highest preference, with an RSCU value of 1.67; for Ala (Alanine), the codon GCU was preferred, with an RSCU value of 1.56; for His (Histidine), the codon CAU was preferred, with an RSCU value of 1.53.

### 3.3. Prediction of RNA Editing Events

**Figure 3:**
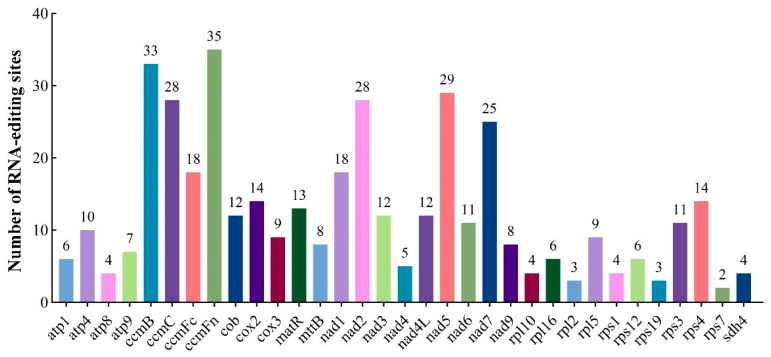
Number of RNA-editing sites of *H. rhamnoides* subsp. *sinensis* mitogenome.

Using the Plant Predictive RNA Editor suite, we successfully identified a total of 411 C-to-U RNA editing sites in 33 mitochondrial PCGs (Figure 3 and Supplementary Table S4). Among these mitochondrial genes, *ccmFn* exhibited the highest number of RNA editing sites, which was 35, followed by *ccmB* with 33 editing sites; genes such as *nad5*, *ccmC*, *nad2*, and *nad7* also showed a relatively high number of editing sites, were 29, 28, 28, and 25, respectively. In contrast, genes including *atp8*, *rpl10*, *rps1*, *sdh4*, *rpl2*, *rps19*, and *rps7* had fewer editing sites, each with four or fewer.

### 3.4. Identification of Repeat Sequences

In addition to the differences in intergenic regions, the widespread presence of repetitive sequences and exogenous fragments also serves as a crucial factor contributing to structural divergence in mitochondrial genomes. The predominant types of repetitive sequences include simple sequence repeats (SSRs), tandem repeats (TRs), and dispersed repeats (DRs). In this study, we identified 176 SSRs, 19 TRs, and 445 DRs (Supplementary Tables S5–S7).

Among the detected SSRs, monomer and tetramer repeats were the most abundant, accounting for 58 (32.95%) and 52 (29.55%), respectively. Dimer and trimer repeats each constituted 17.05% of the total, while pentamer and hexamer repeats represented 2.84% and 0.57%, respectively (Supplementary Table S5). Further analysis of repeat units revealed that A/T mononucleotide repeats were more prevalent than other repeat types. A comparative analysis between *H. rhamnoides* subsp. *sinensis* and eight other plant species demonstrated that SSR abundance ranged from 167 in *H. tibetana* to 404 in *A. membranaceus* (Table 1). The numbers of SSRs in different taxonomic groups were quite different. Specifically, in *Hippophae* (Elaeagnaceae), monomer and dimer repeats accounted for only 47–50% of the total repeats, whereas monomer, dimer, trimer, and tetramer repeats collectively constituted 95–96%. In contrast, in Fabaceae species, monomer and dimer repeats represented over 80% of the total, while monomer, dimer, trimer and tetramer repeats covered 98–100%. Moreover, SSRs in *Hippophae* exhibited a tendency toward longer repeat units compared to those in Fabaceae.

In the mitochondrial genome of *H. rhamnoides* subsp. *sinensis*, we identified 19 tandem repeats, with lengths varying from 9 to 39 base pairs and matching rates exceeding 72.00% (Supplementary Table S6). These results highlighted the diversity and potential functional importance of tandem repeats within the mitochondrial genome.

**Figure 4:**
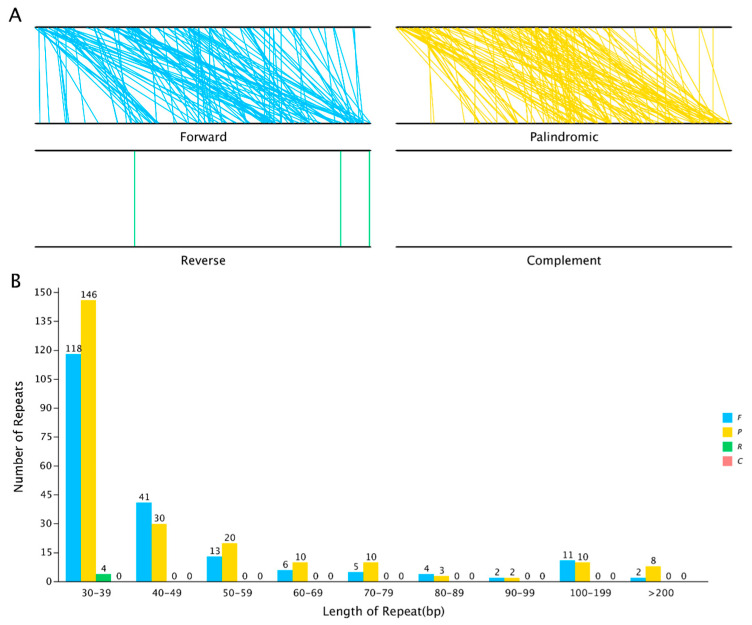
Number of sequence repeats in *H. rhamnoides* subsp. *sinensis.* Note: (**A**) coliner of different type dispersed repeats; (**B**) bar of different type dispersed repeats.

Dispersed repeats, which are scattered throughout the genome, were identified extensively in the mitochondrial genome of *H. rhamnoides* subsp. *sinensis* (Supplementary Table S7). We found 445 dispersed repeats, each having a length of at least 30 bp. Among these, 202 were forward repeats, 239 were reverse repeats and 4 were complement repeats. Collectively, these dispersed repeats spanned 29,642 bp, accounting for 6.52% of the total mitochondrial genome. The length distribution of these repeats showed that short sequences were the most abundant, with only 31 sequences exceeding 100 bp in length, accounting for 6.96% of the total. The longest forward repeat measured 2,839 bp, whereas the longest palindromic repeat reached 4,102 bp (Figure 4).

### 3.5. Ka/Ks Ratio Analysis on PCGs

**Figure 5:**
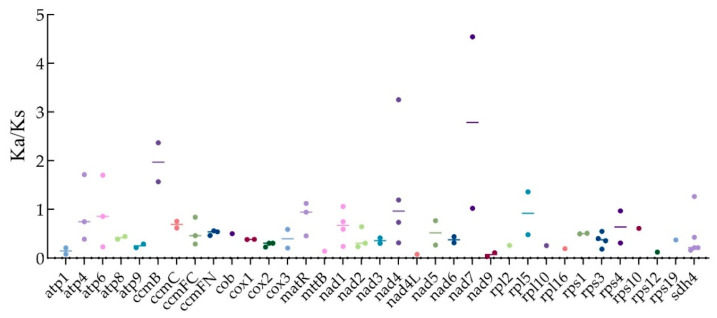
Ka/Ks ratio analysis on PCGs of *H. rhamnoides* subsp. *sinensis.* Note: Dots indicated the same PCGs presence Ka/Ks ratios between *H. rhamnoides* subsp. *sinensis* and other five species. The number of dots for each PCG represented the number of Ka/Ks ratios between five species. The horizontal bar represented the average value of Ka/Ks ratios.

To examine the evolutionary selection pressures acting on mitochondrial PCGs among closely related species, we calculated the ratio of nonsynonymous (Ka) to synonymous (Ks) substitutions (Ka/Ks), as illustrated in Figure 5 and Supplementary Table S8. Comparative analysis between *H. rhamnoides* subsp. *sinensis* and five other species (*F. orientalis*, *Z. jujuba*, *H. gyantsensis*, *H. tibetana*, and *H. salicifolia*) revealed predominant purifying selection. There were 36 orthologous genes shared by five species. However, three genes including *ccmB*, *nad4*, and *nad7*, were under positive selection across all comparison species, while five genes *atp4*, *atp6*, *matR*, *nad1*, and *sdh4* were exhibited positive selection in certain species. Notably, positive selection was more pronounced in distantly related species (*F. orientalis* and *Z. jujuba*). Compared within the *Hippophae* genus, only *nad1* in *H. salicifolia* showed signs of positive selection, whereas all other genes remained under purifying selection.

### 3.6. Homologous Fragments Between Chloroplast and Mitochondrial Genomes

**Figure 6:**
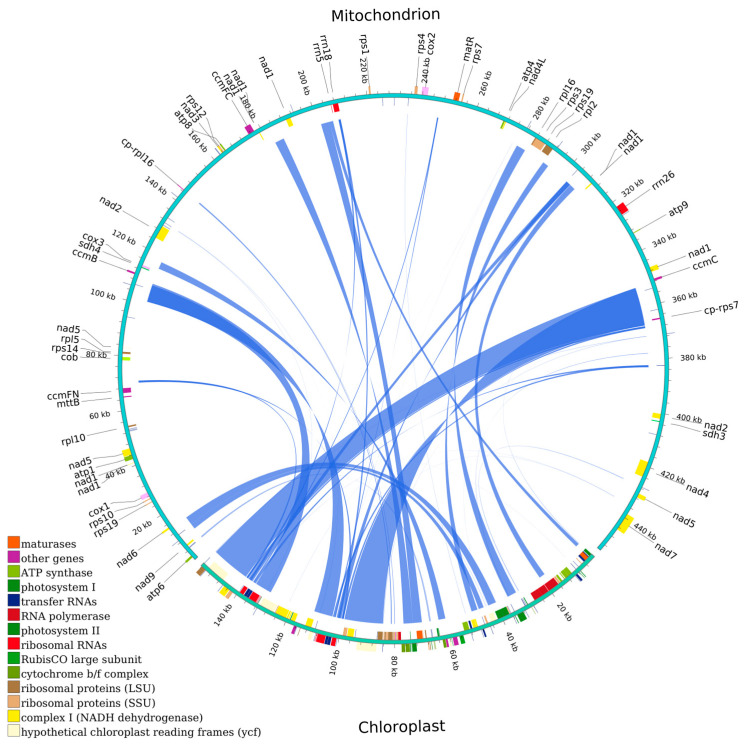
Homologous fragments between chloroplast and mitochondrial genomes.

In the mitochondrial genome of *H. rhamnoides* subsp. *sinensis*, 31 chloroplast-derived fragments were identified, with a total length of 53,910 bp, accounting for 11.86% of the length of mitochondrial genome. The lengths of these homologous fragments ranged from 29 to 14,949 bp. We identified 17 complete chloroplast PCGs within these fragments, namely *cemA*, *clpP*, *ndhB*, *ndhJ*, *petA*, *petB*, *petN*, *psbC*, *psbD*, *psbH*, *psbN*, *psbT*, *rpl23*, *rps4*, *rps7*, *ycf1*, and *ycf2*. In addition, 15 tRNA genes (*tRNA-GUC*, *trnF-GAA*, *trnH-GUG*, *trnI-CAU*, *trnL-CAA*, *trnL-UAA*, *trnM-CAU*, *trnN-GUU*, *trnP-UGG*, *trnR-ACG*, *trnS*-*GGA*, *trnT-GGU*, *trnT-UGU*, *trnV-GAC*, *trnW-CCA*) and 4 rRNA genes (*rrn16*, *rrn23*, *rrn4.5*, *rrn5*) were identified, along with numerous partial genes and intergenic regions (Figure 6).

### 3.7. Synteny Analysis of Mitochondrial Genomes Between H. rhamnoides subsp. sinensis and Related Species

**Figure 7:**
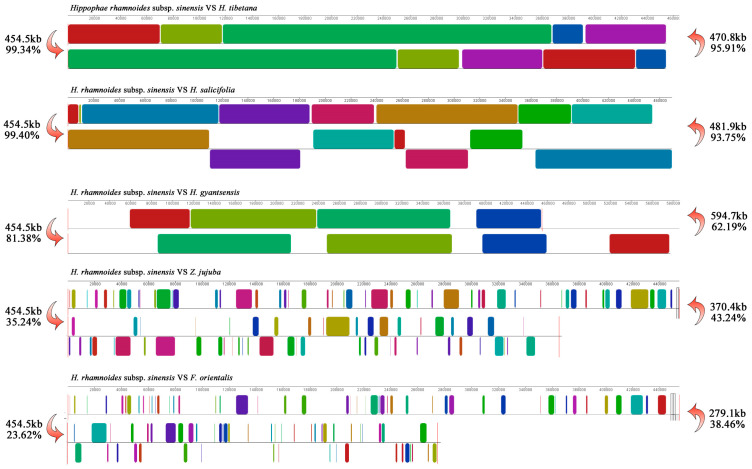
Synteny analysis of the mitochondrial genome in *H. rhamnoides* subsp. *sinensis* and related species. Note: The left-facing arrows indicate the quantity of shared mitochondrial DNA (bp) and percentage between *H. rhamnoides* subsp. *sinensis* and each compared species, while the right-facing arrows display between each compared species and *H. rhamnoides* subsp. *sinensis*. The same color and size represent the same sequence.

Synteny analysis was performed between *H. rhamnoides* subsp. *sinensis* and five other Rosales species to examine conserved regions and structural rearrangements (Figure 7, Supplementary Table S10). The results demonstrated higher sequence similarity and synteny conservation among closely related species. Specifically, *H. rhamnoides* subsp. *sinensis* exhibited 99.34% similarity with *H. tibetana*, 99.40% with *H. salicifolia*, and 81.38% with *H. gyantsensis*. In contrast, its similarity level with *Z. jujuba* was 35.24%, and with *F. orientalis* was 23.62%, indicating lower similarity with distantly related species. The number of root alignment superintervals was 5 between *H. tibetana*, and 78 between *F. orientalis*. The root alignment length was 465,347 bp and 573,224 bp. Through systematic mining of the mitochondrial genome, we identified 15 gene clusters: *cox1–rps10–rps16*, *nad1–atp1–nad5*, *mttB–ccmFN*, *cob–rps14–rpl5*, *sdh4–cox3*, *atp8–rps12–nad3*, *ccmFC–nad1*, *rrn18–rrn5*, *cox2–rps4*, *rps7–matR*, *atp4–nad4L*, *rps3–rpl16*, *rpl2–rps19*, *nad1–ccmC*, and *sdh3–nad2* (Supplementary Table S11). Comparative analysis revealed that *H. rhamnoides* subsp. *sinensis* shared 15, 14, 6, 2, and 8 gene clusters with *H. tibetana*, *H. salicifolia*, *H. gyantsensis*, *F. orientalis*, and *Z. jujuba*, respectively, consistent with synteny patterns. Notably, the *rrn18–rrn5* and *atp4–nad4L* clusters were universally conserved among all six species, indicating high evolutionary stability. The *mttB–ccmFN* and *rpl2–rps19* clusters were unique to the *Hippophae* genus, suggesting their potential utility as taxonomic markers for species identification within this genus.

### 3.8. Phylogenetic Analysis of H. rhamnoides subsp. sinensis

**Figure 8:**
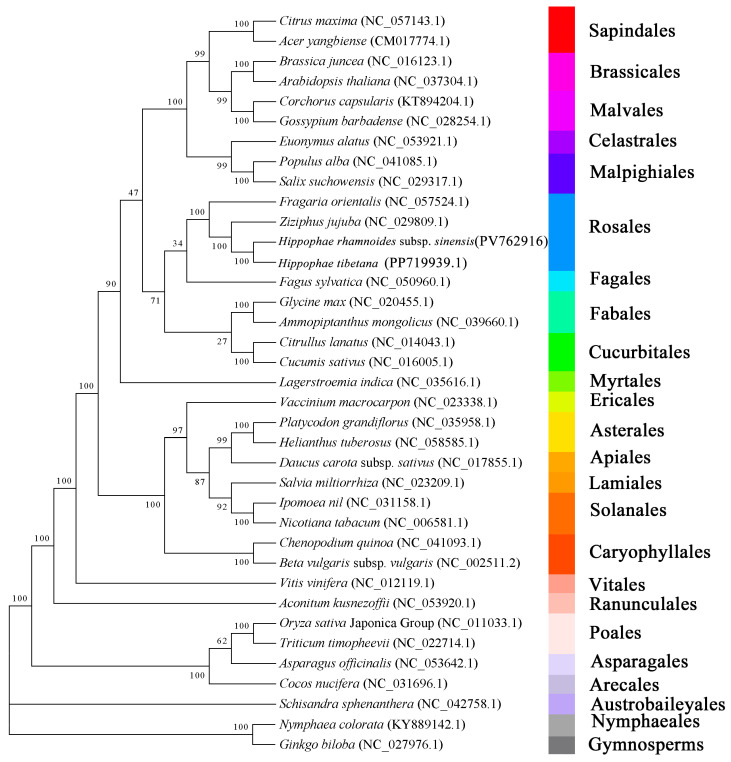
Phylogenetic analysis of *H. rhamnoides* subsp. *sinensis.*

Using the DNA sequences from 29 conserved PCGs, we conducted a phylogenetic analysis involving 37 species (Supplementary Table S1). The specific clustering results and branch support rates were shown in Figure 8. The total aligned length of the phylogenetic tree was 27,257 bp. At the order level, the phylogenetic analysis successfully grouped the selected species without any separation. For example, four Rosales species formed a monophyletic group: *F. orientalis* (NC_057524.1), *Z. jujuba* (NC_029809.1), *H. tibetana* (PP712939.1) and *H. rhamnoides* subsp. *sinensis*; two Sapindales species, *Citrus maxima* (NC_057143.1) and *Acer yangbiense* (CM017774.1) clustered together. At the species level, the two *Hippophae* species formed a well-supported monophyletic group.

## 4. Discussion

### 4.1. Structure and Size of the H. rhamnoides subsp. sinensis Mitochondrial Genome

As the primary site of aerobic respiration in plants, mitochondria play a crucial role in providing energy for cellular activities [36]. They serve as key organelles for plant adaptation to changing environments due to their intricate structure and function, playing dual roles as both a “metabolic hub” and an “environmental sensor” [10,37]. Plant mitochondrial genomes experience significant restructuring resulting in significant variations in structure and size [38] and posing challenges for sequencing and assembly. This study combined second- and third-generation sequencing data, used software for filtering and correction, and then assembled the mitochondrial genome of *H. rhamnoides* subsp. *sinensis* using Unicycler (v0.4.8) with default parameters [39].

The mitochondrial genome of *H. rhamnoides* subsp. *sinensis* was slightly shorter than previously reported for other *Hippophae* species, including *H. gyantsensis* (586,319 bp), *H. salicifolia* (475,105 bp), and *H. tibetana* (464,208 bp) [18,19,20]. Compared with *H. tibetana* and *H. salicifolia*, *H. rhamnoides* subsp. *sinensis* harbored two chloroplast-derived PCGs (*cp–rps7* and *cp–rpl16*), which account for a higher number of PCGs. Additionally, it lacked one tRNA gene (*trnP–TGA*) and one pseudogene (*rps10*), a genomic variation that may confer *H. rhamnoides* subsp. *sinensis* a selective advantage in adapting to a wider range of environmental conditions [18,19]. Non-coding sequences play a significant role in size variations observed in plant mitochondrial genomes [40]. Among these, repetitive sequences are a major component of non-coding DNA and play a crucial role in determining genome size [37]. The presence of repetitive sequences in the *H. rhamnoides* subsp. *sinensis* mitochondrial genome has led to increased complexity of the genome and plays a pivotal role in its rearrangement and evolution [41]. At the same time, it was speculated that DRs were the driving factor responsible for the difference in mitochondrial genome size of *H. rhamnoides* subsp. *sinensis*.

GC content reflects the structural and functional characteristics of genomes, and holds broad significance in evolutionary, biological, and medical research [42]. The mitochondrial genome of *H. rhamnoides* subsp. *sinensis* exhibited a GC content of 44.86%, greater than that of *H. gyantsensis* (44.58%), *H. salicifolia* (44.80%), and *H. tibetana* (44.82%), indicating potential structural stability and adaptation to more diverse environments [43,44]. In this study, the GC contents of *Z. jujuba* and *F. orientalis* both exceeded that of *H. rhamnoides* subsp. *sinensis*, which may contribute to their broader biogeographical distribution. Thus, while GC content is highly conserved within the *Hippophae* genus, there are large differences in mitochondrial genome size.

### 4.2. The Evolution of the H. rhamnoides subsp. sinensis Mitochondrial Genome

The codon usage bias in plant mitochondrial genomes may influence translation efficiency, the stability of mRNA, and gene regulation, indicating the functional specialization and evolutionary pressures of mitochondria within plant cells [45]. This study revealed that most PCGs employ AT-start codons, with leucine, isoleucine, and serine being the most frequent amino acids, consistent with other angiosperms [46,47]. The codon RSCU values >1.00 of the *H. rhamnoides* subsp. *sinensis* mitochondrial genome exhibited codon usage bias towards A/U, which mirrors the typical phenomenon observed in plant mitochondrial genomes [48]. This bias predicted distinct evolutionary trajectories and functional adaptations of *H. rhamnoides* subsp. *sinensis*.

RNA editing is a ubiquitous nucleotide modification process in higher plant mitochondria that increases genetic complexity through flexible and reversible RNA sequence alterations, which in turn enhances plant adaptability to environmental changes and developmental needs [49]. In the mitochondrial genome of *H. rhamnoides* subsp. *sinensis* editing frequency was slightly lower than the 415 sites observed in *H. salicifolia* [19], 448 sites in *M. sativa* [50], and 504 sites in *G. glabra* [51]. The genes *ccmFn*, *ccmB*, *nad5*, *ccmC*, *nad2*, and *nad7* displayed elevated RNA editing densities, consistent with patterns observed in other higher plants [52,53]. These results further elucidate the remarkable evolutionary conservation and close phylogenetic relationships among these PCGs.

Interorganellar gene transfer represents a fundamental driver of dynamic genome evolution in plant cells. This process not only preserves essential organellar functions but also serves as a source of adaptive genetic variation [54,55]. In the mitochondrial genome of *H. rhamnoides* subsp. *sinensis*, 31 chloroplast-derived fragments were identified, accounting for 11.86% of the length of the mitochondrial genome. This chloroplast-to-mitochondrion transfer frequency was markedly lower than in *H. tibetana*, with 41 fragments covering 17.97% [18], and yet it marginally exceeded the 30 fragments comprising 2.10% observed in *Ilex metabaptista* [56]. The presence of two chloroplast-derived transcripts, *cp–rps7* and *cp–rpl16*, constituted a primary factor contributing to the elevated mRNA abundance in the mitochondrial genome of *H. rhamnoides* subsp. *sinensis* compared with other *Hippophae* species. These transferred genes are functionally associated with diverse physiological processes including ion transport, photosynthetic carbon metabolism, protein quality control, photorespiration, and cyclic electron transport, suggesting potential adaptations to more heterogeneous ecological niches.

The Ka/Ks ratio is commonly used to estimate the rate of functional evolution in genomes and to reveal molecular mechanisms underlying adaptive evolution [57]. Most PCGs exhibit high evolutionary conservation, with Ka/Ks ≤ 1. The ratio greater than 1 (Ka/Ks > 1) suggests positive selection, indicating adaptive evolutionary changes [58]. In this study, we compared the Ka/Ks ratios among *H. rhamnoides* subsp. *sinensis*, *H. gyantsensis*, *H. salicifolia*, *H. tibetana*, *Z. jujuba*, and *F. orientalis*. Eight mitochondrial genes, including *atp4*, *atp6*, *ccmB*, *matR*, *nad1*, *nad4*, *nad7*, and *sdh4*, showed signatures of positive selection (Ka/Ks > 1), with most being functionally associated with energy metabolism. Positive selection signals were detected in the mitochondrial genome of *H. tibetana*, specifically affecting the *atp6*, *ccmB*, *nad4L*, and *nad7* genes [18]. Similarly, in *H. salicifolia*, the *ccmB*, *nad4L*, and *nad4* genes exhibited signatures of positive selection pressure [19]. *H. rhamnoides* subsp. *sinensis* exhibited a higher number of positively selected genes compared to other *Hippophae* species, suggesting it may face greater environmental pressures and stronger selective forces.

Synteny analysis of plant mitochondrial genomes reveals both conserved essential functions and evolutionary innovation potential, highlighting their significant applications in environmental adaptation and crop trait improvement [59]. In this study, we performed synteny analysis of the *H. rhamnoides* subsp. *sinensis* mitochondrial genome with related Rosales species. The observed similarity aligned with the phylogenetic positions shown in the cladogram (Figure 8) and largely corresponded to Lian Yongshan’s taxonomic framework for *Hippophae* [1]. It further demonstrated substantial mitochondrial genome sequence diversity among Rosales species. In contrast, all 15 gene clusters were conserved between *H. rhamnoides* subsp. *sinensis* and *H. tibetana*, whereas only the *rrn18–rrn5* and *atp4–nad4L* gene clusters, which are widely conserved in plants were also found in *F. orientalis* [60]. These results corroborated the phylogenetic relationships inferred from cladogram and synteny analyses, consistently supporting their evolutionary divergence patterns.

Plant mitochondrial genomes are characterized by structural dynamism, integration of exogenous sequences, and complex regulatory mechanisms. Their evolutionary strategies balance conservative preservation of respiratory functions with radical recombination frequency, establishing them as pivotal tools in plant taxonomy, evolutionary biology, population genetics, and comparative genomics [37]. In this study, we constructed a phylogenetic tree based on mitochondrial *H. rhamnoides* subsp. *sinensis* PCGs and 36 other plant species. Four species from Rosales, two species from Sapindales, two species from Solanales, and two species from Cucurbitales formed coherent groupings, further validating the accuracy of our phylogenetic reconstruction. Simultaneously, the results revealed close phylogenetic affinities among Rosales, Fagales, Fabales, Malpighiales, and Celastrales, corroborating the congruence with established systematic classifications. These findings robustly supported the utility of mitochondrial genome data in elucidating plant taxonomic relationships.

## 5. Conclusions

The *H. rhamnoides* subsp. *sinensis* mitogenome comprised 454,489 bp, encoding 40 PCGs, 3 rRNA genes, 30 tRNA genes, and 2 pseudogenes, which displayed a pronounced AT bias. The mitochondrial genome’s complexity was augmented by the incorporation of chloroplast-derived *cp-rps7* and *cp-rpl16* genes, along with four copies of *trnM-CAT* and three copies of *trnP-TGG*. In *H. rhamnoides* subsp. *sinensis* mitochondrial genome codon usage bias among those with RSCU values >1.00 exhibited a distinct A/U bias and 411 C-to-U RNA editing sites across 33 PCGs were identified. The higher editing sites were observed in *ccmFn*, *ccmB*, *nad5*, *ccmC*, *nad2*, and *nad7* genes. The genome contained 31 chloroplast-derived sequences, representing 11.86% of its total genomic content. *H. rhamnoides* subsp. *sinensis* mitochondrial energy metabolism-related genes *ccmB*, *nad4L*, and *nad7* had undergone positive selection pressure evolution compared with other Rosales species. Extensive syntenic conservation was observed between *H. rhamnoides* subsp. *sinensis* and its congeneric species, while lower conservation was detected with *Z. jujuba* and *F. orientalis*. *H. rhamnoides* subsp. *sinensis* and *H. tibetana* shared all the 15 gene clusters, while only two clusters *rrn18*-*rrn5* and *atp4*-*nad4L* were conserved with *F. orientalis*. Phylogenetic analysis demonstrated close evolutionary affinities between Rosales (containing *H. rhamnoides* subsp. *sinensis*), Fagales, Fabales, Malpighiales, and Celastrales, affirming the value of mitochondrial genomic data in elucidating plant phylogenetic relationships. This study produced a high-resolution mitochondrial genome sequence for *H. rhamnoides* subsp. *sinensis*, offering fundamental insights into its biogeographic distribution patterns and providing an evolutionary framework for implementing molecular breeding strategies in the *Hippophae* genus.

## Figures and Tables

**Table 1:** Comparison of mitogenome dispersed repeats in *H. rhamnoides* subsp. *sinensis* and eight other species.

Species	Total Number of Repeats	Total Length of Repeats (bp)	Genome Size (bp)	Proportion in Genome (%)
*Astragalus membranaceus*	77	13,583	398,048	3.4
*Caragana spinosa*	90	19,585	378,373	5.2
*Glycyrrhiza glabra*	127	13,272	440,064	3.0
*Medicago sativa*	68	13,140	290,285	4.5
*Oxytropis arctobia*	88	10,943	343,007	3.2
*Pisum fulvum*	88	14,041	379,906	3.7
*Trigonella foenum-graecum*	137	37,659	345,604	10.9
*H. rhamnoides* subsp. *sinensis*	445	29,642	454,489	6.52
*H. tibetana*	257	31,757	464,208	6.84

## Data Availability

To support the results, the mitochondrial genome data have been deposited in the NCBI database, with login accession number PV762916.

## Supplementary Materials

The following supporting information can be downloaded at: https://www.mdpi.com/article/10.3390/plants14142170/s1. Supplementary Figure S1A: *H.* subsp. *sinensis* tree; Supplementary Figure S1B: *H.* subsp. *sinensis* with fruits; Supplementary Table S1: species list of phylogenetic analysis; Supplementary Table S2: function genes; Supplementary Table S3: Rscu; Supplementary Table S4: rna edit; Supplementary Table S5: SSRs; Supplementary Table S6: tandem; Supplementary Table S7: dispersed; Supplementary Table S8: Ka/Ks; Supplementary Table S9: cp_mt; Supplementary Table S10: synteny analysis; Supplementary Table S11: gene clusters.
