# Supplementary figures and images for: Assembly and Analysis of the Mitochondrial Genome of Hippophae rhamnoides subsp. sinensis, an Important Ecological and Economic Forest Tree Species in China
Source: Plants (Basel). 2025 Jul 14;14(14):2170. doi: 10.3390/plants14142170 (PMC12299680; doi:10.3390/plants14142170)

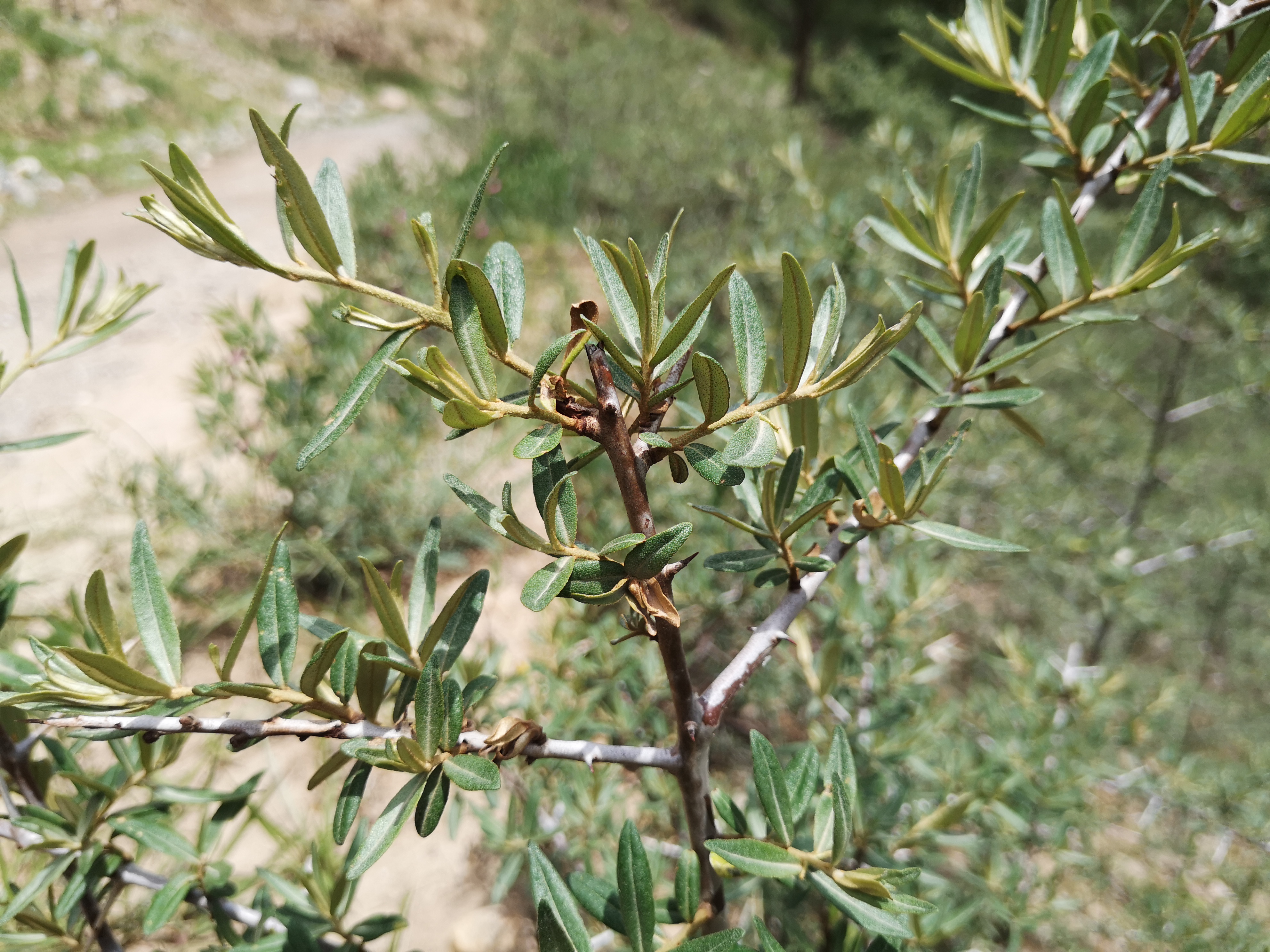

Supplement: Supplementary file 1 [file plants-14-02170-s001.zip › Supplementary Figure 1 A H. subsp. sinensis tree.jpg]

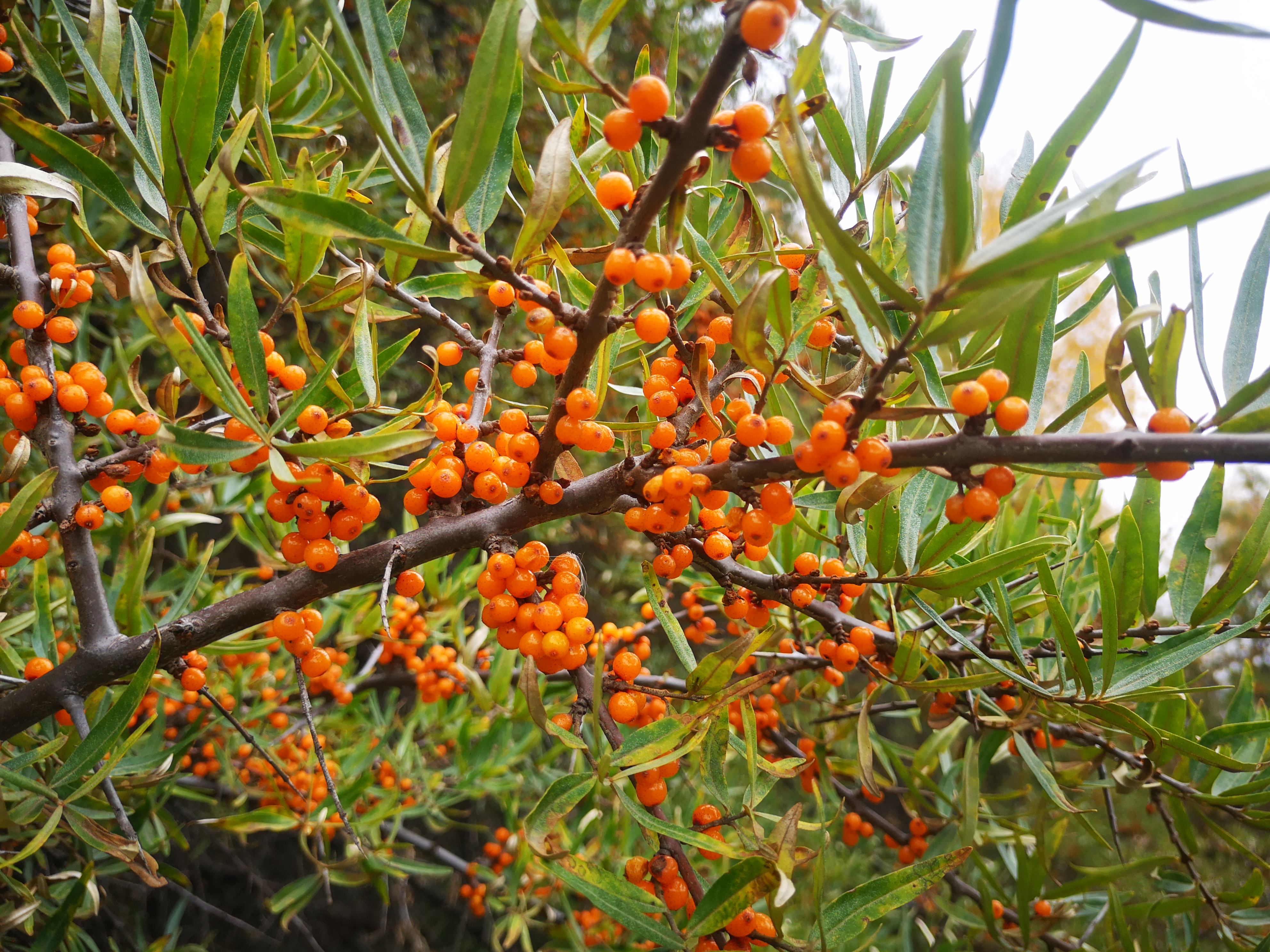

Supplement: Supplementary file 1 [file plants-14-02170-s001.zip › Supplementary Figure 1 B H. subsp. sinensis with fruits.jpg]
